# Tracheomalacia and Interstitial Pneumonia in a 95-Year-Old Woman With Undiagnosed Rheumatoid Arthritis: A Case Report

**DOI:** 10.7759/cureus.87205

**Published:** 2025-07-02

**Authors:** Natsumi Yamamoto, Shiho Amano, Kohei Oka, Chiaki Sano, Ryuichi Ohta

**Affiliations:** 1 Community Care, Unnan City Hospital, Unnan, JPN; 2 Community Medicine Management, Shimane University Faculty of Medicine, Izumo, JPN

**Keywords:** autoimmune diseases, collagen disease-related interstitial pneumonia, elderly, family medicine, general medicine, immunosuppressive therapy, prednisolone, rheumatoid arthritis, tracheomalacia

## Abstract

This case report describes a 95-year-old woman with a history of cardiovascular and gastrointestinal diseases who presented with fever and anorexia. She was diagnosed with influenza A and secondary bacterial pneumonia. Despite antiviral and antibiotic treatment, her respiratory condition worsened. Detailed examination revealed characteristic joint deformities, elevated rheumatoid factor, and anti-cyclic citrullinated peptide antibodies. Imaging showed interstitial pneumonia and paradoxical breathing with elevation of the membranous portion of the trachea, indicating tracheomalacia. The patient was diagnosed with previously unrecognized rheumatoid arthritis with associated interstitial pneumonia and tracheomalacia. Immunosuppressive therapy with prednisolone and azathioprine improved her condition. This case highlights the importance of thorough systemic evaluation in elderly patients with unexplained respiratory symptoms and the need to consider late-onset autoimmune diseases, including rheumatoid arthritis, in the differential diagnosis.

## Introduction

Rheumatoid arthritis (RA) is a chronic systemic autoimmune disease that primarily targets synovial joints, resulting in persistent inflammation, progressive joint destruction, and long-term disability [1]. Beyond the joints, RA has a broad spectrum of extra-articular manifestations, which can significantly contribute to patient morbidity and mortality. According to [2], approximately 40% of patients with RA develop extra-articular complications during the disease course, with the respiratory system being the most frequently involved. Pulmonary manifestations are diverse and may include interstitial lung disease, bronchiectasis, pleural effusion, and, less commonly, airway abnormalities [3,4]. Among these, tracheomalacia, defined by abnormal collapsibility and weakening of the tracheal wall, is a rare but potentially life-threatening condition in RA patients [5]. Its clinical recognition is challenging, as symptoms such as dyspnea, wheezing, or recurrent respiratory infections can be mistakenly attributed to more common pulmonary complications [5].

Elderly-onset RA, which develops after the age of 60, often presents atypically and can be underdiagnosed, particularly in very old adults [6]. Extra-articular manifestations may predominate over joint symptoms in this population, leading to diagnostic delays and suboptimal management [6]. Moreover, comorbidities and age-related physiological changes can further complicate the presentation and outcomes of the disease [6]. Here, we present a rare case of previously undiagnosed RA in a 95-year-old woman who developed both tracheomalacia and interstitial pneumonia, likely precipitated by an influenza infection, underscoring the diagnostic challenges and clinical significance of extra-articular RA manifestations in the elderly.

## Case presentation

A 95-year-old woman presented to a rural hospital with fever, sore throat, and anorexia. She had lived independently one week ago, when she had a mild fever up to 37°C and a mild dry cough. Three days before the admission, her appetite decreased, and systemic muscular pain worsened, impinging on her movement. On admission day, her activities of daily living (ADLs) were independent. She lived with her daughter and visited her community center weekly for recreation and joy. She had no exposure to infected people and had no travel history or exposure to wild animals. She had a medical history of angina, hypertension, dyslipidemia, reflux esophagitis, osteoporosis, chronic bronchitis, constipation, and insomnia. Her medications included nicorandil of 15 mg, benidipine of 2 mg, pravastatin of 10 mg, vonoprazan of 10 mg, eldecalcitol of 0.5μg, magnesium oxide of 1500 mg, and eszopiclone 1 mg daily.

On admission, the patient's vital signs were as follows: body temperature 37.8°C, blood pressure 150/84 mmHg, heart rate 91 beats per minute, respiratory rate 28 breaths per minute, and peripheral oxygen saturation (SpO₂) 90% on room air. Physical examination revealed paradoxical breathing (seesaw pattern), wheezing, fine crackles at the bases of both lungs, and erythema of the oropharynx. The metacarpophalangeal (MCP) joints exhibited ulnar deviation, suggesting chronic RA. Laboratory testing showed elevated C-reactive protein (CRP) and lactate dehydrogenase (LDH) levels, along with mild hypoalbuminemia (Table 1).

**Table 1:** Initial laboratory data of the patient CRP, C-reactive protein

Parameter	Level	Reference
White blood cells	6.8	3.5–9.1 × 10^3^/μL
Neutrophils	77.1	44.0–72.0%
Lymphocytes	12.6	18.0–59.0%
Hemoglobin	11.0	11.3–15.2 g/dL
Hematocrit	32.7	33.4–44.9%
Mean corpuscular volume	95.4	79.0–100.0 fl
Platelets	18.3	13.0–36.9 × 10^4^/μL
Total protein	7.8	6.5–8.3 g/dL
Albumin	2.9	3.8–5.3 g/dL
Total bilirubin	0.5	0.2–1.2 mg/dL
Aspartate aminotransferase	46	8–38 IU/L
Alanine aminotransferase	26	4–43 IU/L
Lactate dehydrogenase	288	121–245 U/L
Blood urea nitrogen	11.4	8–20 mg/dL
Creatinine	0.6	0.40–1.10 mg/dL
Serum Na	127	135–150 mEq/L
Serum K	4.2	3.5–5.3 mEq/L
Serum Cl	92	98–110 mEq/L
CRP	2.92	<0.30 mg/dL
Urine test	-	-
Leukocyte	Negative	Negative
Protein	Negative	Negative
Blood	Negative	Negative
Influenza antigen test	A(+)	-
	B(-)	-

An influenza antigen test was positive for type A virus.

**Figure 1:**
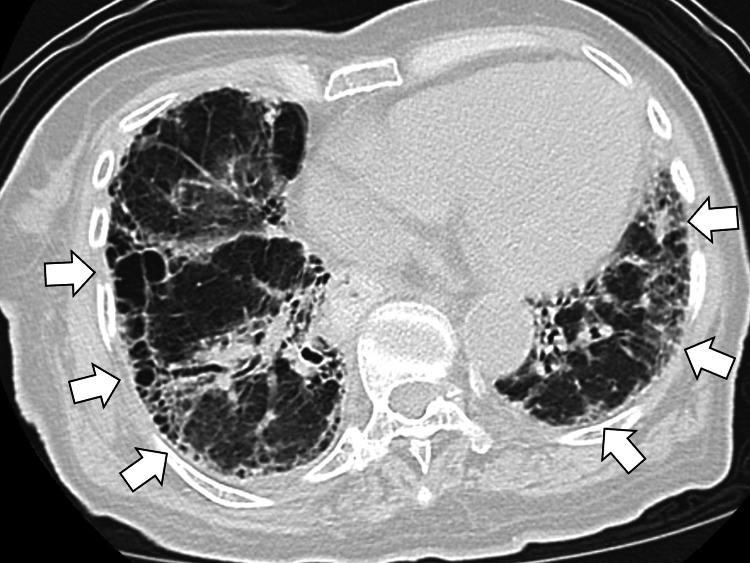
Chest computed tomography revealing a pattern consistent with nonspecific interstitial pneumonia, including reticular and ground-glass opacities along the bronchovascular bundles and honeycombing at the lung bases (white arrows)

Chest radiography demonstrated bilateral ground-glass opacities. Chest computed tomography (CT) revealed a pattern consistent with nonspecific interstitial pneumonia (NSIP), including reticular and ground-glass opacities along the bronchovascular bundles and honeycombing at the lung bases (Figure 1).

An influenza A infection with secondary bacterial pneumonia was diagnosed, and treatment was initiated with oseltamivir phosphate and ceftriaxone.

Despite antiviral and antibiotic therapy, the patient’s dyspnea and paradoxical breathing persisted. Autoantibody testing showed markedly elevated rheumatoid factor (RF; 243 IU/mL) and anti-cyclic citrullinated peptide (anti-CCP) antibodies (>500 U/mL). Antinuclear antibody (ANA), myeloperoxidase anti-neutrophil cytoplasmic antibody (MPO-ANCA), proteinase 3 anti-neutrophil cytoplasmic antibody (PR3-ANCA), and anti-aminoacyl-tRNA synthetase (anti-ARS) antibodies were all negative. Serum levels of Krebs von den Lungen-6 (KL-6) were elevated (Table 2).

**Table 2:** Additional laboratory data of the patient Rheumatoid factor and ACPA levels were markedly elevated, consistent with autoimmune pathology. ANA was borderline positive. Both proteinase 3-ANCA (PR3-ANCA) and myeloperoxidase-ANCA (MPO-ANCA) were within normal limits, ruling out ANCA-associated vasculitis. Anti-aminoacyl tRNA synthetase (anti-ARS) antibody was negative. KL-6 was elevated, suggesting interstitial lung involvement. Serum IgG was elevated, indicating polyclonal hypergammaglobulinemia, while IgA and IgM levels were within or near the upper limit of normal. The T-SPOT test for tuberculosis was negative. ACPA, anti-cyclic citrullinated peptide antibody; ANA, antinuclear antibody; ANCA, anti-neutrophil cytoplasmic antibody; ARS, aminoacyl-tRNA synthetase; KL-6, Krebs von den Lungen-6; MPO, myeloperoxidase; PR3, proteinase 3; T-SPOT, T-cell spot test for tuberculosis; Ig, immunoglobulin

Parameter	Level	Reference
Rheumatoid factor	243	<15 IU/mL
ACPA	≧500	<5 U/mL
Antinuclear antibody	40	<40
PR3-ANCA	<1.0	<3.5 U/mL
MPO-ANCA	<1.0	<3.5 U/mL
anti-ARS antibody	<5.0	<25
KL-6	795	105-401 U/mL
IgG	2002	870-1700 mg/dL
IgA	420	110-410 mg/dL
IgM	107	35-220 mg/dL
T-SPOT	Negative	Negative

**Figure 2:**
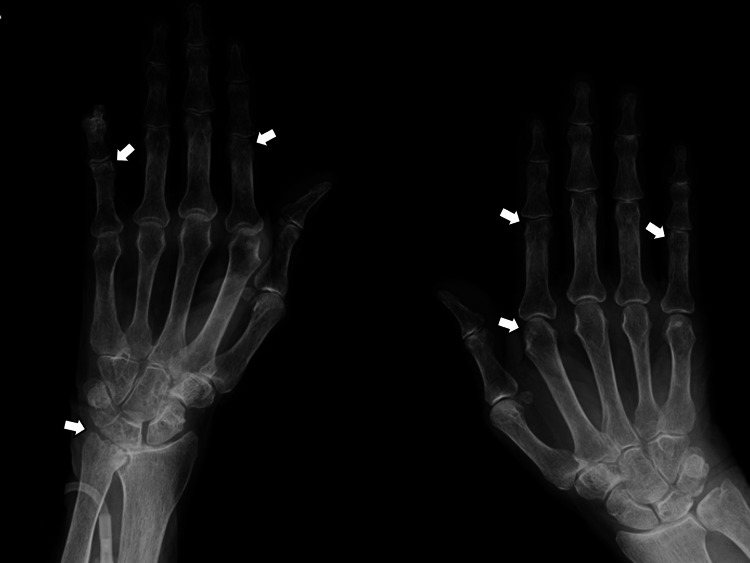
Radiography of the hands revealing bony erosions, consistent with rheumatoid arthritis (white arrows)

Radiography of the hands revealed bony erosions, consistent with RA (Figure 2).

**Figure 3:**
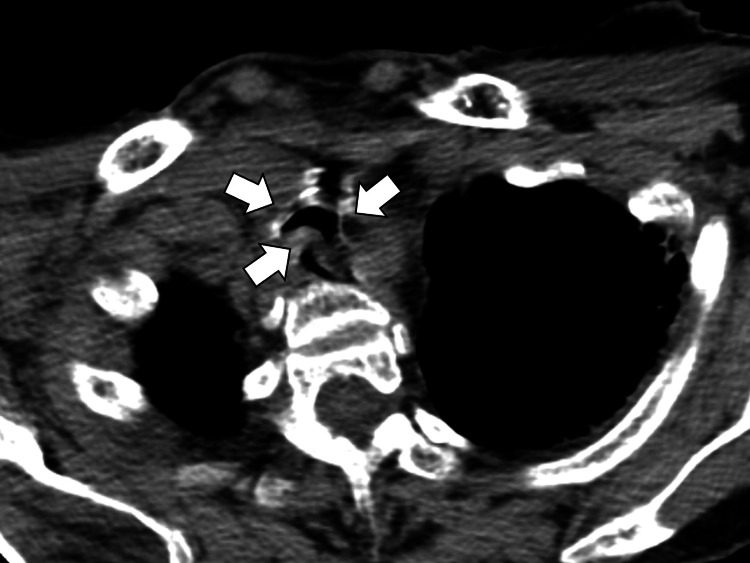
Chest computed tomography demonstrating the elevation of the membranous portion of the main bronchi during inspiration, which suggested tracheomalacia (white arrows)

A follow-up chest CT demonstrated elevation of the membranous portion of the main bronchi during inspiration, which suggested tracheomalacia (Figure 3).

Based on these findings, the patient was diagnosed with RA complicated by interstitial pneumonia and tracheomalacia. Treatment with oral prednisolone (30 mg/day) and azathioprine (25 mg/day) improved oxygenation and reduced inflammatory markers. Corticosteroid tapering was initiated, and the patient was transferred to a rehabilitation ward to continue recovery.

## Discussion

In this case, we encountered a 95-year-old woman without a prior diagnosis of RA who developed worsening respiratory distress due to interstitial pneumonia following an influenza infection. A comprehensive physical examination revealed signs of advanced RA and secondary tracheomalacia. Early initiation of immunosuppressive therapy, including high-dose prednisolone, led to clinical remission [7,8]. This case highlights the importance of thorough systemic assessment in elderly patients with progressive symptoms. It underscores the need to consider extra-articular complications when RA is diagnosed at an advanced stage. Multidisciplinary management should be prioritized in such scenarios.

Elderly-onset RA is often underdiagnosed and may present primarily with extra-articular manifestations that exacerbate the patient's general condition [9]. Seropositive RA, especially with high RF titers, is associated with more aggressive joint disease and a higher incidence of extra-articular involvement compared to seronegative cases [10]. High RF titers are frequently linked to complications such as interstitial lung disease and vasculitis in older individuals and may predict poor prognosis [11].

In the present case, the patient had not been previously diagnosed with RA, yet physical examination revealed clear evidence of joint swelling and deformities. These findings, along with serological and radiographic data, facilitated the diagnosis of RA and its associated complications. Delays in diagnosis are common in elderly patients, especially in rural areas, where healthcare-seeking behaviors may differ and access to specialized care is limited [12]. This case underscores the importance of conducting a complete systemic examination and considering coexisting autoimmune diseases in elderly patients with progressive symptoms.

Tracheomalacia may complicate elderly-onset RA when the diagnosis is delayed. In adults, tracheomalacia is predominantly acquired and may result from various causes, including chronic obstructive pulmonary disease (COPD), relapsing polychondritis, trauma, obesity, or external compression [13]. Relapsing polychondritis is frequently associated with other connective tissue diseases, including RA [14]. In this patient, the respiratory condition deteriorated following an influenza infection, which led to the diagnosis of both interstitial pneumonia and tracheomalacia. Retrospective evaluation of joint imaging and serological markers confirmed longstanding, undiagnosed RA as the underlying cause.

Delayed diagnosis of RA in remote areas remains a significant clinical issue [6,12,15]. As the disease progresses, the likelihood of developing diverse complications increases. When encountering progressive symptoms in patients with known or suspected RA, clinicians should consider early, aggressive, and multidisciplinary treatment approaches, including high-dose corticosteroids, to improve clinical outcomes [16].

## Conclusions

Through this case, we considered the possibility that persistent dyspnea following treatment for bacterial pneumonia secondary to influenza infection may signal an underlying, previously undiagnosed condition such as rheumatoid arthritis and its complications, including interstitial pneumonia and tracheomalacia. This highlights the importance of maintaining a broad differential diagnosis when managing respiratory symptoms in elderly patients. When respiratory function fails to improve as expected after treatment of infectious diseases, clinicians should be vigilant for the presence of an underlying autoimmune disease, which may not only account for the persistent symptoms but also contribute to progressive respiratory decline. Early recognition and appropriate management of such conditions are crucial to improving outcomes and preventing further complications in this vulnerable population.
